# Supplementary figures and images for: SARM1 detection in myelinating glia: sarm1/Sarm1 is dispensable for PNS and CNS myelination in zebrafish and mice
Source: Front Cell Neurosci. 2023 Apr 5;17:1158388. doi: 10.3389/fncel.2023.1158388 (PMC10113485; doi:10.3389/fncel.2023.1158388)

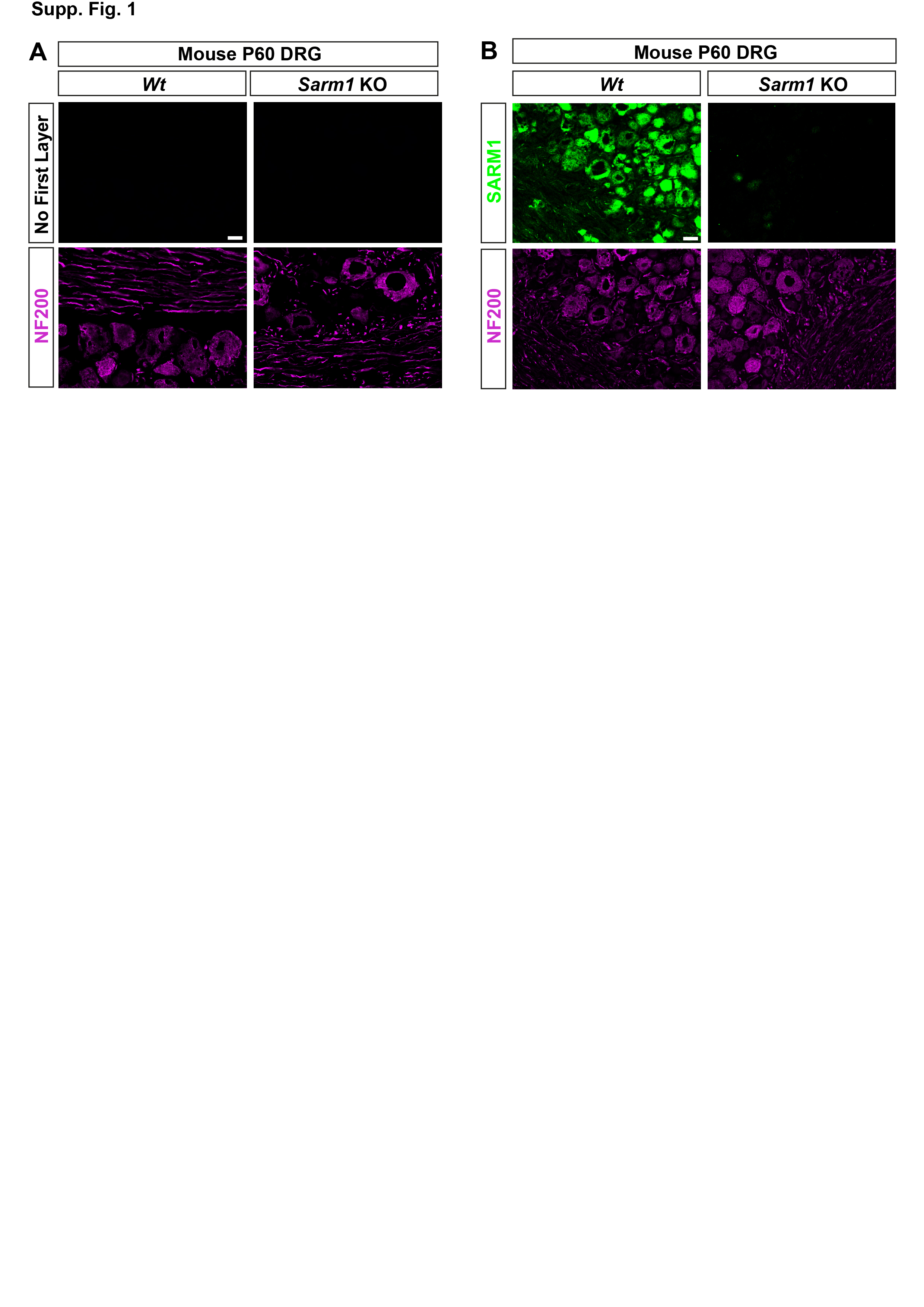

Supplement: Supplementary Figure 1 — No first layer SARM1 antibody controls. (A) Representative immunofluorescence images of transverse sections of Wt and Sarm1 KO mouse dorsal root ganglia (DRGs) to show lack of SARM1 (blue) signal in the absence of addition of primary antibody. NF-200 (neurofilament light chain; magenta) to show neurons. Scale bar 25 μm. (B) Representative immunofluorescence images of transverse sections of Wt and Sarm1 KO mouse dorsal root ganglia (DRGs) to show lack of SARM1 (blue) signal in the KO sample in the presence the primary antibody. The Wt panel shows co-localization of NF-200 (magenta) positive neurons with SARM1 (blue). Scale bar 25 μm. [file Image_1.JPEG]

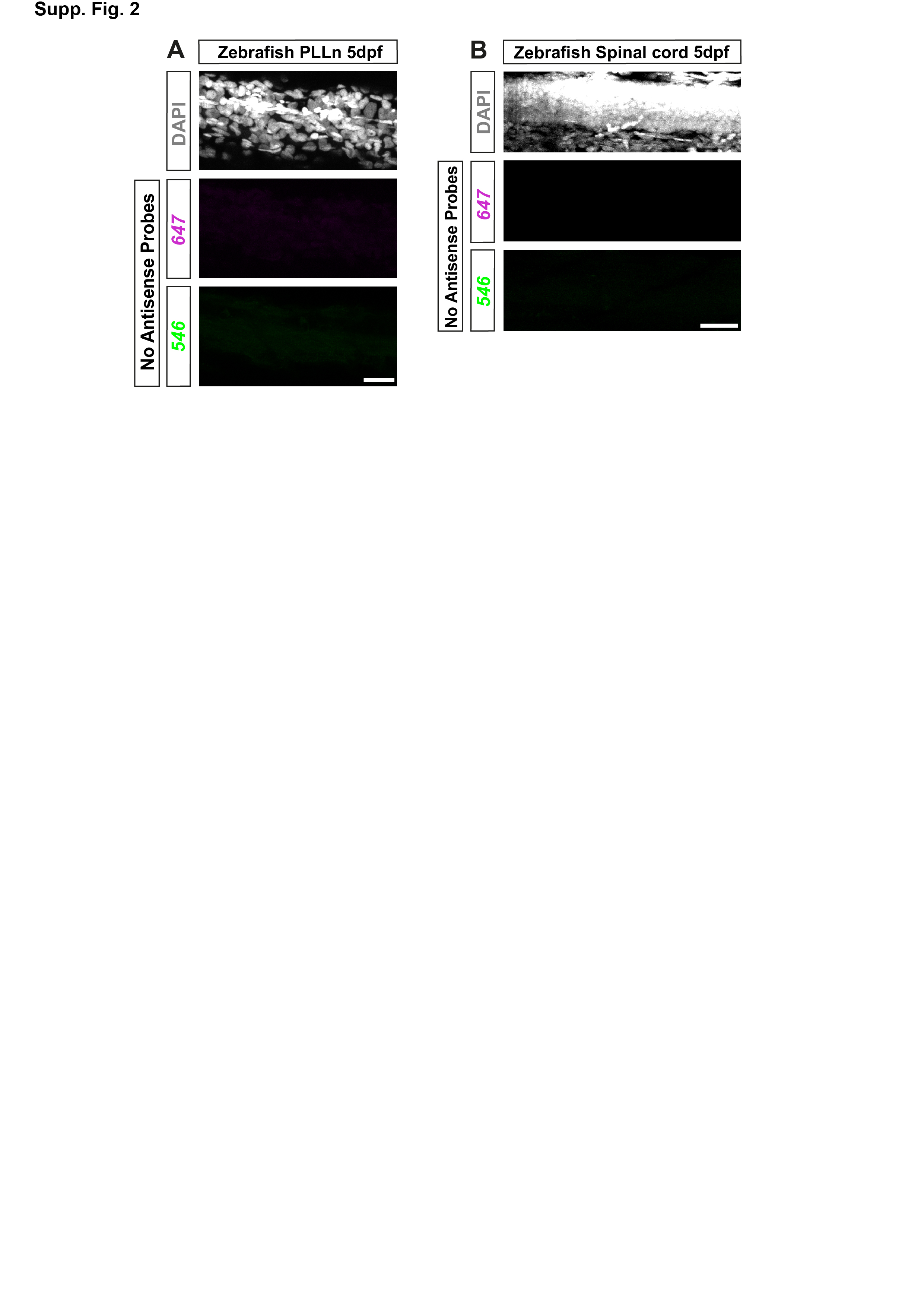

Supplement: Supplementary Figure 2 — sarm1 HCR no first layer controls. (A) MAX projection, lateral view of the PLLn of a 5dpf larva with no probes (no first layer control) to sox10 nor sarm1 applied. Images show DAPI (gray), and hairpins tagged to Alexa647 (magenta) and Alexa546 (green). Scale bar 25 μm. (B) MAX projection, lateral view of the spinal cord of a 5dpf larva with no antisense probes to sox10 nor sarm1 applied. Images show DAPI (gray), and hairpins tagged to Alexa647 (magenta) and Alexa546 (green). Scale bar 25 μm. [file Image_2.JPEG]

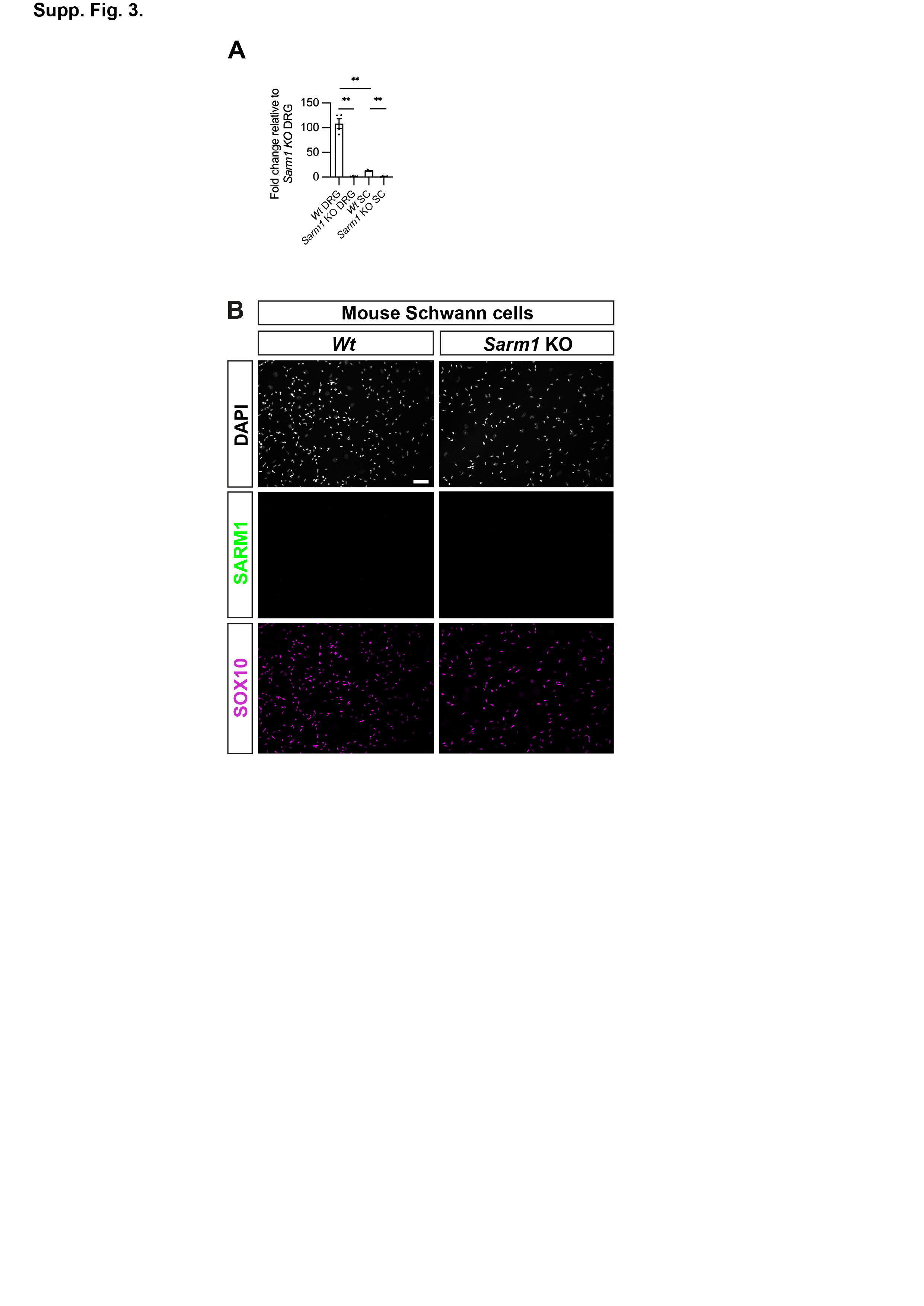

Supplement: Supplementary Figure 3 — Sarm1 mRNA but not SARM1 protein is detectable in mouse Schwann cells. (A) qPCR analysis of Sarm1 expression in cultured mouse Wt and Sarm1 KO DRGs and Wt and Sarm1 KO Schwann cells [SC, n = 4 (4 biological replicates: 4 sciatic nerves from 2 animals pooled per biological replicate); **p < 0.01]. (B) Freshly plated Wt and Sarm1 KO, P2 dissociated mouse Schwann cells both have undetectable levels of SARM1 protein (green). Cultures labelled for DAPI (gray) and SOX10 (magenta) (n = 3: two-four mice each from 3 L producing three separate cultures). Scale bar 50μm. [file Image_3.JPEG]

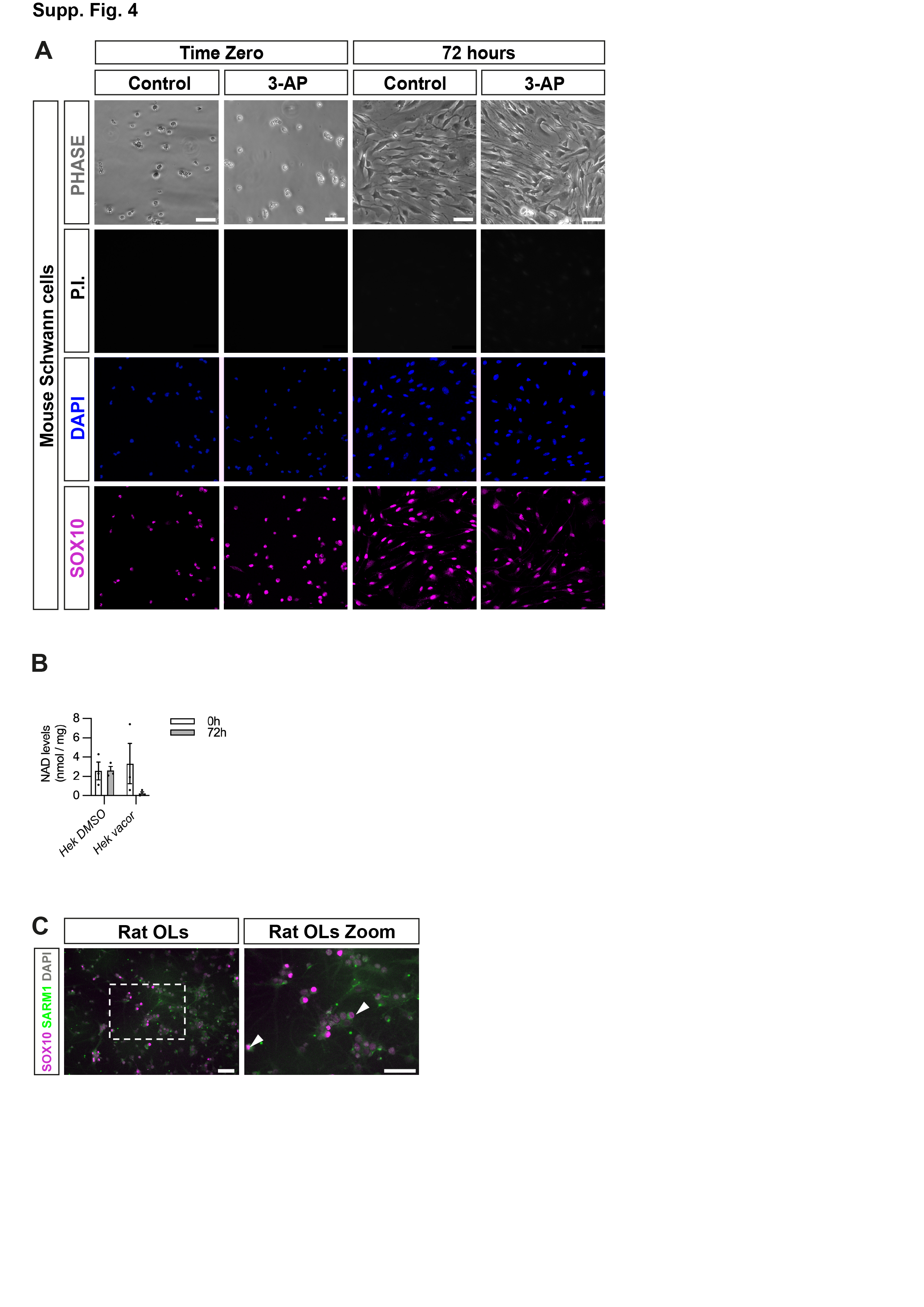

Supplement: Supplementary Figure 4 — Cultured Schwann cells are insensitive to 3-AP induced cell death. (A) Dissociated and freshly plated P2 mouse Schwann cells cultured in defined medium with water or 250 mM 3-AP for 72 h show no cell death, judged by propidium iodide (P.I) and DAPI nuclear staining and SOX10 immunocytochemistry, 101.6 ± 1.7% SOX10 positive cells survived after 72 h 250 mM 3-AP treatment (mean and SEM, n = 3: two-four mice, each from 3 L producing three separate cultures, p = 0.68). Scale bar 25 μm. (B) Intracellular NAD+ levels decrease in HEK293 cells when treated for 72 h with 100 mM vacor. (C) Cultured rat oligodendrocytes (SOX10 positive; magenta) express SARM1 protein (green) using a commercial polyclonal anti-SARM1 antibody. Zoomed in area (indicated by dashed box). Arrowheads indicate SOX10 positive SARM1 positive cells. Scale bars 50 μm. [file Image_4.JPEG]

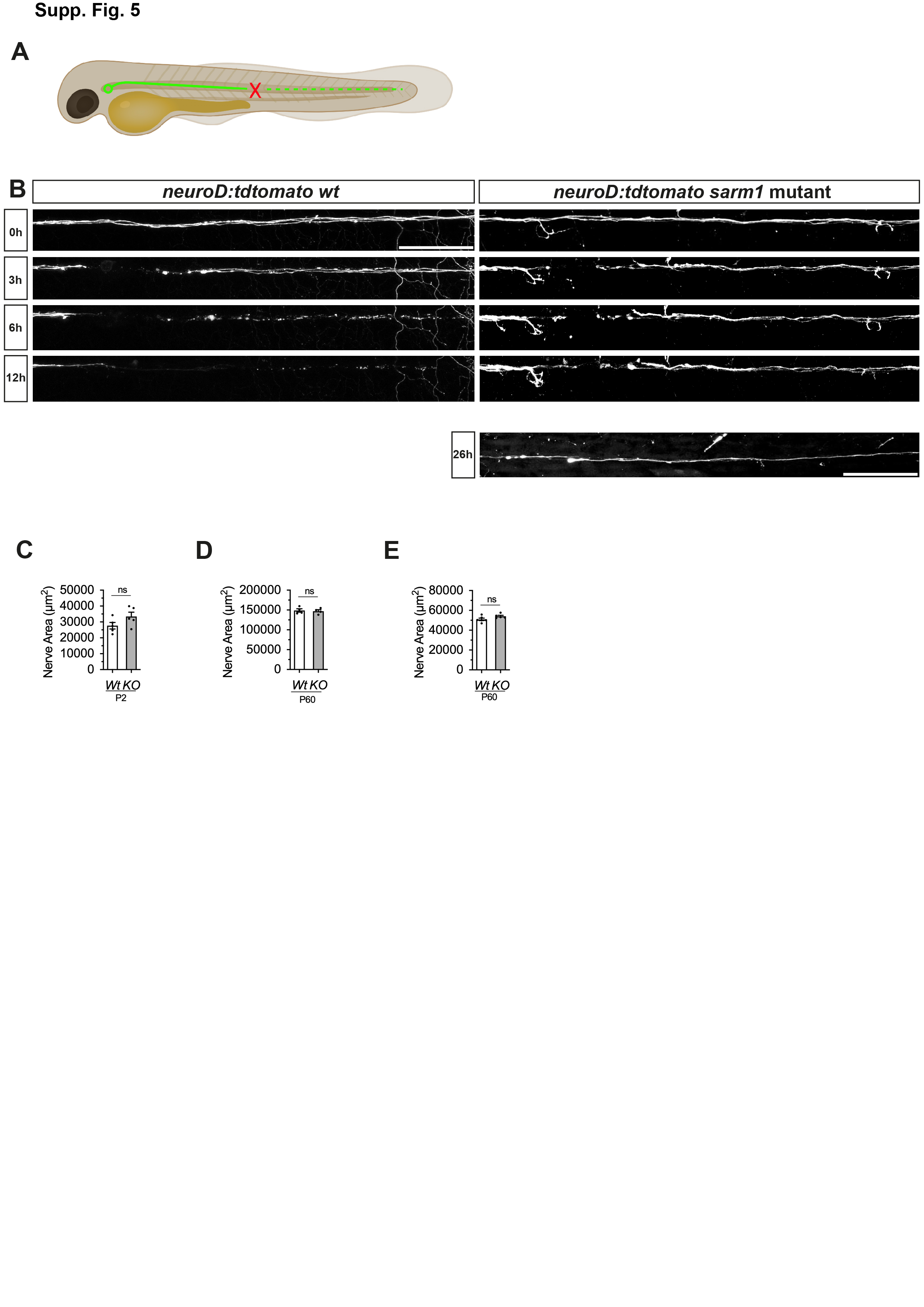

Supplement: Supplementary Figure 5 — Axon degeneration after PLLn laser axotomy is substantially delayed in absence of functional Sarm1 in zebrafish larvae. (A) Schematic of overview of 2-photon laser axotomy of the PLLn (green) in larval zebrafish. (B) wt and sarm1SA11193/SA11193 (sarm1 mutant) fish were injected at the one cell zygote stage with neuroD:tdtomato DNA constructs and 2-photon laser axotomy was performed in both genotypes at 4dpf. While wt PLLn axons start to degenerate around 3 h and are completely degenerated at 6 h post axotomy, PLLn axons in sarm1 mutant larvae remain intact at least up until 26 h post imaging (imaging stopped due to UK home office regulations) (n = 5). Scale bar 100 μm. (C) The mean total nerve area of P2 mouse sciatic nerves in Wt, 27,674 ± 2,050 μm2 and Sarm1 KO, 33,527 ± 2,648 μm2, is not significantly different (mean and SEM, n = 5; p = 0.1508). (D) Per nerve profile, the total nerve area of both Wt (148,879 ± 4,209 μm2) and Sarm1 KO (147,181 ± 3,619 μm2) nerves is very similar (n = 4; p = 0.8857). (E) Per nerve profile, the total nerve area of both Wt (51,002 ± 1,238 μm2) and Sarm1 KO (54,130 ± 982.6 μm2) optic nerves were not significantly different (mean and SEM, n = 6 WT; n = 5 KO; p = 0.1255). [file Image_5.JPEG]
